# Mirrizi Syndrome and Markedly Elevated Levels of Carbohydrate Antigen 19-9 in the Absence of Malignant Disease

**DOI:** 10.1155/2017/2416901

**Published:** 2017-04-26

**Authors:** Natasha Shah, Eula Tetangco, Hafiz Muhammad Sharjeel Arshad, Hareth Raddawi

**Affiliations:** University of Illinois at Chicago/Christ Hospital Advocate Medical Center, Oak Lawn, IL, USA

## Abstract

Elevated carbohydrate antigen 19-9 (CA19-9) beyond 1000 U/L occurs in nonneoplastic conditions which is causing questioning of the use of CA19-9 as a marker for screening. We report a case where a 51-year-old male with Mirrizi Syndrome (MS) presented with markedly increased CA19-9 level (4,618 U/mL). MS is a rare complication characterized by compression of the common bile or hepatic duct caused by an impacted gallstone in the cystic duct or neck of the gallbladder. Biliary epithelial cells secrete CA19-9: it is hypothesized that increased proliferation of such cells caused by inflammation leads to increased secretion. CA19-9 should not be used as a diagnostic tool, but rather for surveillance.

## 1. Introduction

Carbohydrate antigen 19-9 (CA19-9) is a mucinic glycoprotein that is expressed on pancreatic and biliary duct cells. CA19-9 has been the tumor marker of choice for assisting in the detection of pancreatic, biliary, and gastric carcinoma [1–3]. The diagnosis of pancreatic cancer has been extremely difficult despite imaging modalities including CT and ERCP, and several biochemical markers, including carcinoembryonic antigen (CEA), pancreatic ribonuclease, and various peptide hormones, have failed to produce any significant relationship with the disease. However since the early 1980s, clinical investigations showed CA19-9 to be of significance in differentiating pancreatic cancer [3, 4]. Normally, only trace amounts of the protein are found in the serum, but in the setting of pancreatic or biliary cancer, serum levels rise exponentially, though there is no direct correlation in serum concentration and tumor burden [1]. CA19-9 levels are used for preoperative staging, pancreatic biliary cancer prognosis, assessment of tumor resectability, and diagnosis of tumor recurrence [5]. The upper normal limit of CA19-9 is 37 U/mL. Levels above this cutoff have a sensitivity of 90 percent and specificity of 45 percent for malignant biliary obstruction due to pancreatic or biliary neoplasm. With levels above 200 U/mL, sensitivity is 65 percent and specificity 91 percent. With levels rising higher than 1000 U/mL, the specificity increases to 100 percent which has made CA19-9 the current gold standard tumor marker assessment in diagnosis and prognosis of upper gastrointestinal, pancreatic, and biliary cancer [4, 6].

However, multiple case reports and studies have shown that elevated CA19-9 levels beyond 1000 U/L occur in nonneoplastic benign conditions which lead to questioning of the use of CA19-9 as an adequate tumor marker for screening. An overlap of elevated values has been found in cancer and noncancer patients [5]. An increase in CA19-9 levels has been found to be in organ-specific diseases including the following: acute and chronic pancreatitis, cholelithiasis, cholecystitis, achalasia, acute hepatitis, hepatic cirrhosis, respiratory diseases, and systemic diseases such as diabetes mellitus and rheumatic and autoimmune disorders. Nonneoplastic liver conditions including liver cysts, severe steatosis, autoimmune hepatitis, chronic alcoholic hepatitis, and hepatic cirrhosis [7] have been associated with high CA19-9 levels. In noncholestatic liver disease patients, high levels of CA19-9 have been found to be highly specific for severe liver fibrosis [8].

Among nonmalignant diseases, obstructive jaundice is the most frequent condition associated with increases in CA19-9. CA19-9 levels are three times higher than the upper normal limit, which raises concerns for malignant disease [9]. CA19-9 concentration greater than 10,000 U/mL has been reported in patients with biliary stones. A dramatic decline in CA19-9 levels is seen after the obstruction is resolved [10]. We present a case of a 51-year-old male who suffered from a rare condition, Mirrizi Syndrome, that presented with a markedly increased CA19-9 level above 1000 U/mL. No evidence of pancreatic biliary or gastric carcinoma was found and a dramatic drop in CA19-9 levels was seen after intervention. Without the persistence of elevated CA19-9 markers, we hypothesized that the levels were elevated due to the obstruction and any injury induced to the liver secondary to alcohol use. Similar to other authors, this case demonstrates that CA19-9 markers should not be used as a guaranteed diagnostic tool, but rather a tool for surveillance [5].

## 2. Case Report

A 51-year-old male with past medical history of alcohol abuse was admitted to the hospital after presenting with 10-day history of mid epigastric abdominal pain and progressive jaundice with dark colored urine. The patient reported feeling unwell 2 weeks prior with early satiety and anorexia. The patient noted yellow discoloration of his sclera, skin, and pruritus, along with dark colored urine and clay like stools. He had experienced a 10-pound weight loss within 6 weeks. He denied nausea, vomiting, fevers, sweats, chills, or dysphagia. On physical examination, patient was found to have icteric sclera with few spider angiomata on the upper anterior chest. The patient had an enlarged liver on exam with palpable right lower lobe. Hemoccult was negative with clay colored stools.

On admission, blood tests showed a total bilirubin of 18 mg/dL (direct 17.3), alkaline phosphatase 306 U/L, aspartate aminotransferase (AST) 167 U/L, alanine aminotransferase (ALT) 497 U/L, Gamma-Glutamyl Transferase (GGTP) 1,180 U/L, and white blood cell count 5000 *μ*L. Patient was admitted with suspicion of biliary obstructive jaundice and for further workup. Computed tomography scan performed the same day showed cholelithiasis with additional stones extending down into the distal cystic duct with visible dilation of the common bile duct above the insertion of the cystic duct. Intrahepatic ductal dilation was also seen. Serum CA 19-9 was 4,618 U/L (normal 0–37 U/mL) and CEA was 2.4 (normal 0–2.5 mcg/L).

An initial endoscopic ultrasound (EUS) and endoscopic retrograde cholangiopancreatography (ERCP) showed a 14 mm stone in the cystic duct with upstream dilation of the cystic duct and common hepatic duct. No obvious pancreatic head mass was visualized. Patient underwent a magnetic resonance cholangiopancreatogram (MRCP) that confirmed the pancreatic parenchyma appeared normal without any masses or lobularity. The location of the 11 × 12 × 15 mm common bile duct calculi was confirmed to be at the confluence of the cystic and common hepatic duct. Four days after the initial procedure, patient underwent a repeat ERCP. In the meanwhile, patient's bilirubin had increased to 21 mg/dL and he still appeared icteric with complaints of pruritus.

Repeat ERCP findings suggested benign ectasia of the common hepatic duct as the presence of the stone in the cystic duct was not found to be occluding the bile duct. There was no evidence of stricture. This led to suspicion of intrahepatic cholestasis with a contributing element of alcoholic liver disease. A 5 French 7 cm double pigtail stent was placed into the common hepatic duct to prevent any effect of any cystic duct stones on the bile duct. A 50 French 4 cm single pigtail pancreatic protective stent was placed into the pancreatic duct. Postprocedure cholangiogram showed no filing defects. After sphincterotomy and biliary stent placement, the cystic duct was patent. The cytology brushing from the bile duct was negative for malignancy. There was a subsequent dramatic drop in bilirubin (9.6 mg/dL) and liver enzymes after biliary sphincterotomy and biliary stent placement. One month after discharge, the patient returned for an elective cholecystectomy. At surgery, biopsy revealed evidence of marked chronic inflammation with areas of acute inflammation consistent with cholecystitis. Pathology reports showed cholelithiasis with mucosal ulceration but there was no visible tumor or findings consistent with malignancy. CA19-9 levels were checked when the patient was seen for follow-up as an outpatient, and the levels had significantly dropped and was found to be within normal limits.

## 3. Discussion

Mirrizi Syndrome (MS) is a benign but rare complication characterized by compression of the common bile duct or common hepatic duct caused by an impacted gallstone in the cystic duct or neck of the gallbladder. This complication causes obstructive jaundice due to the direct compression by the stone or due to fibrosis as a result of chronic inflammation. Reported incidence is between 0.05 and 5.7 percent in patients undergoing cholecystectomy. MS can be diagnosed with MRCP as was done in our case: a stone was visualized in the cystic duct [11].

MS and other benign conditions that cause biliary obstruction have been found to be associated with exponentially high CA19-9 levels. A similar case was reported in the Israel Medical Journal Association where a level of CA19-9 over >23,000 was found in patient with grade 1 Mirrizi Syndrome that normalized after a >1.5 cm stone was stone removed [12]. Another case of obstructive jaundice secondary to an impacted stone in the common bile duct causing cholangitis was associated with a CA19-9 level > 61,000 U/mL. The patient was followed postoperatively for 1 year, and no imaging studies showed evidence of malignancy [13]. Suspicion for malignancy in patients who present with obstructive jaundice and high levels of CA19-9 is warranted as the differential diagnosis includes gallbladder cancer and pancreatic cancer; however, CA19-9 levels should be monitored after the obstruction is removed as these levels normalize. Patients with carcinoma have levels that tend to remain persistently high. In a study of 60 patients with biliary obstruction, it was found that almost all patients with benign conditions had a drop in CA19-9 levels after intervention [5].

It is unclear as to what causes the elevation in CA19-9 levels in the setting of hyperbilirubinemia [12]. Biliary epithelial cells secrete CA19-9; thus it is hypothesized that increased proliferation of biliary epithelial cells in the setting of obstructive jaundice caused by irritation, inflammation, and bile stasis leads to increased secretion and accumulation of CA19-9 [5]. Increased permeability between the bile and blood leads to an accumulation of CA19-9 in the serum. The resolution of the cholestasis or obstruction will reverse this process and a decrease in CA19-9 levels will be seen [9]. CA19-9 is likely hepatically cleared and excreted in the bile; thus intrahepatic congestion or extrahepatic biliary obstruction can lead to rising levels of CA19-9 [1].

This case demonstrates a high CA19-9 level secondary to Mirrizi Syndrome, though it can be assumed based on review of literature that the patient's concomitant alcohol induced liver disease may have been a contributing factor in regard to the high levels of CA19-9 seen in this case. Several cases have demonstrated levels of CA19-9 higher than 1000 in the setting of alcohol liver disease. In all cases, no evidence of malignancy was observed. In 1996, the European Journal of Gastroenterology and Hepatology published four unique cases with patients who had markedly elevated CA19-9 levels and 2 patients with levels above >1000 which was due to alcoholic biopsy proven cirrhosis with no concomitant pancreatic disease [2]. A German case report presents a 58-year-old male who presented with jaundice and weight loss. Unlike our patient, he had no biliary pathology contributing to his symptoms. He was found to have a CA19-9 level greater than 10,000 U/mL. Liver biopsy was consistent with alcoholic liver disease. No additional imaging studies showed any evidence or concern for malignancy. After ceasing alcohol intake, the patient's clinical presentation improved and biochemical abnormalities normalized. Three months after initial presentation, CA19-9 level was found to be at 96 U/mL [5].

CA19-9 is normally absent in the liver; however tissue damage can induce CA19-9 synthesis as tissue inflammatory damage promotes fibrotic tissue deposition and parenchymal regeneration [7]. This is supportive of CA19-9 markers being highly specific for liver fibrosis [8]. Immunohistochemical analysis has shown high CA19-9 reactivity in hepatic inflammatory areas, specifically in bile ductile cells and hepatocytes in ductular metaplasia [7]. In the setting of liver disease, CA19-9 levels need to be interpreted cautiously as liver disease can contribute to false elevations.

## 4. Conclusion

CA19-9 in the setting of obstructive jaundice or liver disease is not useful in identifying malignancy as it can cause false elevations above the cutoff as demonstrated in this case. It is important that the serum level result be interpreted in accordance with the patient's medical history, physical examination, additional labs, and imaging studies. Though this may seem like a setback, CA19-9 levels can be monitored in addition to bilirubin and liver transaminases as a way of monitoring disease progression [1]. Discontinuation of an offending agent, such as virus, toxin, medication, or in this case stone obstruction, will show improvement in liver and biliary disease which is characterized by the rapid decrease in CA19-9 after a short period of time. This case demonstrates a rare case seen in the United States that is similar to cases seen in European countries where biliary obstruction has been found to be associated with high levels of CA19-9 and after the obstruction was alleviated, the levels began to rapidly decline. Understanding and being aware that high levels of CA19-9 can be present without concurrent malignancy can prevent unnecessary invasive diagnostic workup.

## References

[1] Maestranzi S., Przemioslo R., Mitchell H., Sherwood R. A. (1998). The effect of benign and malignant liver disease on the tumour markers CA19-9 and CEA. *Annals of Clinical Biochemistry*.

[2] Mathurin P., Cadranel J.-F., Bouraya D. (1996). Marked increase in serum CA 19-9 level in patients with alcoholic cirrhosis: report of four cases. *European Journal of Gastroenterology and Hepatology*.

[3] Safi F., Beger H. G., Bittner R., Büchler M., Krautzberger W. (1986). CA 19-9 and pancreatic adenocarcinoma. *Cancer*.

[4] Steinberg W. (1990). The clinical utility of the CA 19.9 tumor-associated antigen. *Case Reports in Gastrointestinal Medicine*.

[5] Marrelli D., Caruso S., Pedrazzani C. (2009). CA19-9 serum levels in obstructive jaundice: clinical value in benign and malignant conditions. *American Journal of Surgery*.

[6] De Goede E., Yap S. (1997). An exceptional high concentration of serum CA 19.9 in a patient with alcoholic liver disease Department of Hepatology, University Hospital Leuven. *Gut*.

[7] Bertino G., Ardiri A. M., Calvagno G. S. (2013). Carbohydrate 19.9 antigen serum levels in liver disease. *BioMed Research International*.

[8] Schöniger-Hekele M., Müller C. (2006). The combined elevation of tumor markers CA 19-9 and CA 125 in liver disease patients is highly specific for severe liver fibrosis. *Digestive Diseases and Sciences*.

[9] Ventrucci M., Pozzato P., Cipolla A., Uomo G. (2009). Persistent elevation of serum CA 19-9 with no evidence of malignant disease. *Digestive and Liver Disease*.

[10] Basso D., Meggiato T., Fabris C. (1992). Extra-hepatic cholestasis determines a reversible increase of glycoproteic tumour markers in benign and malignant diseases. *European Journal of Clinical Investigation*.

[11] Milone M., Musella M., Maietta P. (2014). Acute acalculous cholecystitis determining Mirizzi syndrome: case report and literature review. *BMC Surgery*.

[12] Gibor U., Perry Z. H., Netz U. (2015). CA 19-9 in the presence of obstructive jaundice due to mirizzi syndrome. *Israel Medical Association Journal*.

[13] Peterli R., Meyer-Wyss B., Herzog U. (1999). CA19-9 has no value as a tumor marker in obstructive jaundice. *Case Reports in Gastrointestinal Medicine*.

